# Influence of Isolation Temperature on Isolating Diverse Lactic Acid Bacteria from Kimchi and Cultural Characteristics of Psychrotrophs

**DOI:** 10.4014/jmb.2303.03047

**Published:** 2023-05-12

**Authors:** Hye In Ko, Chang Hee Jeong, Se-Jin Park, So-Rim Kim, Jong-Bang Eun, Tae-Woon Kim

**Affiliations:** 1 Technology Innovation Research Division, World Institute of Kimchi, Gwangju 61755, Republic of Korea; 2 Department of Integrative Food, Bioscience and Biotechnology, Chonnam National University, Gwangju 61186, Republic of Korea; 3 Honam National Institute of Biological Resources, Mokpo 587262, Republic of Korea

**Keywords:** Kimchi, lactic acid bacteria, psychrotrophs, isolation temperature

## Abstract

Kimchi is a traditional Korean fermented vegetable that is stored and fermented at low temperatures. However, kimchi lactic acid bacteria (LAB) are typically isolated under mesophilic conditions, which may be inappropriate for isolating the diverse LAB. Therefore, this study investigated the suitable conditions for isolating various LAB from kimchi. Here, LAB were isolated from four kimchi samples using MRS, PES, and LBS media and varying isolation temperatures (30, 20, 10, and 5°C). Then, MRS was selected as the suitable medium for LAB isolation. A comparison of culture–dependent and culture–independent approaches indicated that 5°C was not a suitable isolation temperature. Thus, the number and diversity of LAB were determined at 30, 20, and 10°C using 12 additional kimchi samples to elucidate the effect of isolation temperature. With the exception of two samples, most samples did not substantially differ in LAB number. However, *Leuconostoc gelidum*, *Leuconostoc gasicomitatum*, *Leuconostoc inhae*, *Dellaglioa algida*, *Companilactobacillus kimchiensis*, *Leuconostoc miyukkimchii*, *Leuconostoc holzapfelii*, and *Leuconostoc carnosum* were isolated only at 10 and 20°C. The growth curves of these isolates, except *Leu. holzapfelii* and *Leu. carnosum*, showed poor growth at 30°C. This confirmed their psychrotrophic characteristics. In *Weissella koreensis*, which was isolated at all isolation temperatures, there was a difference in the fatty acid composition of membranes between strains that could grow well at 30°C and those that could not. These findings can contribute to the isolation of more diverse psychrotrophic strains that were not well isolated under mesophilic temperatures.

## Introduction

Kimchi is a traditional Korean food made by fermenting various vegetables. It is produced via fermentation of salted vegetables with various spices and ingredients, including garlic, red pepper powder, ginger, green onion, and salt-fermented seafood. Although there are various microorganisms derived from several raw materials, lactic acid bacteria (LAB) dominate kimchi during the fermentation process [1]. The most common LAB genera in kimchi are *Leuconostoc*, *Lactobacillus*, *Weissella*, *Lactococcus*, and *Pediococcus* [1, 2]. In general, the dominant species are *Leuconostoc mesenteroides*, *Leu. citreum*, *Leu. gasicomitatum*, *Leu. carnosum*, *Leu. gelidum*, *Lactilactobacillus sakei*, *W. koreensis*, and *Weissella cibaria* [1, 3]. The microbial community in kimchi varies depending on the fermentation stage. Various microbial communities exist in the early and middle stages of fermentation, with *Leu. mesenteroides* as the predominant species [2]. Dominant species at the late stage of fermentation include *Lactiplantibacillus plantarum*, *L. sakei*, and *W. koreensis* [1]. The microbial community affects the sensory properties of kimchi, which are attributable to the different metabolisms and metabolites of each strain [4]. These microbial community changes occur as the pH decreases because of organic acid produced by LAB during kimchi fermentation [5].

Temperature is also one of the critical factors affecting the microbial community in kimchi, owing to the differences in the optimal cultural temperature among bacterial strains [6‒9]. Hence, the fermentation temperature of kimchi influences its LAB flora, leading to a difference in the kimchi sensory characteristics [1, 10].

Kimchi is generally prepared at home during early winter, kept at room temperature for 1‒2 days, and stored at approximately 0°C to inhibit excessive fermentation. Alternatively, it is fermented at 4°C until consumption [11, 12]. Furthermore, commercially available kimchi is generally stored below 10°C for distribution [13]. When it is shipped to the consumer, the refrigerated truck temperature is maintained between 0‒5°C [13]. Then, consumers store and ferment the commercial kimchi at approximately 0°C before consumption [13]. These kimchi fermentation conditions serve as the bacteria cultural environment, making them an important part of the strain isolation process.

In a previous study, a LAB strain was isolated from yogurt at a medium temperature (37°C), which is the appropriate cultural temperature for these strains (32‒45°C) [14, 15]. Furthermore, thermophilic bacteria were cultured at a high temperature (50°C) [17]. In addition, a high salinity medium is used to isolate halophilic microbial species from high salt environments [16]. This is a fundamental prerequisite for isolating strains. Desirable conditions are required to successfully isolate microbes from kimchi, with consideration for its fermentation temperature.

Extensive isolation and identification of more LAB strains from kimchi could considerably contribute to our understanding of the role of each species in the kimchi fermentation process. Although low temperatures are suitable environments for some species of *Leuconostoc* belonging to psychrotrophic LAB [1, 3, 18], the current methods for isolating LAB from kimchi usually apply mesophilic isolation conditions (30°C) [19, 20]. Hence, it is necessary to investigate the suitable conditions to isolate more diverse LAB from kimchi.

In this study, LAB species that are isolated from kimchi based on the isolation temperature and culture medium were identified. In addition, the culture characteristics of strains that were isolated at low temperatures and exhibited poor growth under mesophilic conditions were confirmed.

## Materials and Methods

### Preparation and Physicochemical Properties of Kimchi Samples

Kimchi samples were obtained from homemade kimchi (HM) and seven local processing plants in Korea. Here, the name of the company where the kimchi was purchased is used as the sample name (Kimchi-town (KT), Han-sang-gung (HSG), Tae-seo (TS), Tae-baek (TB), Ye-so-dam (YSD), I-nam-jang (INJ), Ggot-soon-yi (GSY)). KT was fermented at 4°C for 29 days and sampling was done on Days 1 (KT 1), 15 (KT 2), and 30 (KT 3) to confirm suitable isolation conditions. Next, kimchi samples were fermented at 10°C (10HSG, 10TS, 10TB, 10YSD, 10INJ, and 10GSY) and 4°C (4HSG, 4TS, 4TB, 4YSD, 4INJ, and 4GSY) for 1 and 2 weeks, respectively. Each sample was subsequently ground and filtered through sterile gauze to obtain kimchi soup. The pH was measured using a pH meter (STARA1117, Thermo Fisher Scientific Inc., USA). The titratable acidity was calculated in terms of lactic acid using 0.1 N NaOH until the sample pH reached 8.3. Finally, soluble solid contents and salinity of the kimchi samples were measured using a refractometer (PAL-1, Atago Co., Japan) and a salt meter (PAL-ES2, Atago Co.).

### Isolation and Identification of LAB from Kimchi

To investigate the appropriate isolation condition range of LAB from kimchi, the kimchi soup of HM, KT 1, KT 2, and KT 3 were serially diluted and spread on De Man Rogosa Sharp (MRS), phenylethyl alcohol sucrose (PES, peptone 5.0 g/l; yeast extract 0.5 g/l; sucrose 20.0 g/l; ammonium sulfate 2.0 g/l; magnesium sulfate haptahydrate 0.5 g/l; potassium dihydrogen phosphate 1.0 g/l; agar 15.0 g/l), and *Lactobacillus* selection (LBS, pancreatic digest of casein 10.0 g/l; yeast extract 5.0 g/l; dextrose 20.0 g/l; monopotassium phosphate 6.0 g/l; ammonium citrate 2.0 g/l; sodium acetate hydrate 25.0 g/l; magnesium sulfate 0.575 g/l; manganese sulfate 0.12 g/l; ferrous sulfate 0.034 g/l; Tween 80 1.0 g/l; agar 15.0 g/l) agar. The media were then incubated at 30, 20, 10, and 5°C for 24‒48, 48‒72, 144‒168, and 336–360 h, respectively. In addition, samples were spread on plate count agar (PCA, tryptone 5.0 g/l; yeast extract 2.5 g/l; dextrose 1.0 g/l; agar 15.0 g/l) and incubated at 35°C for 24–48 h. To confirm suitable isolation temperatures for isolating diverse LAB, 10INJ, 10GSY, 10YSD, 10HSG, 10TB, 10TS, 4INJ, 4GSY, 4YSD, 4HSG, 4TB, and 4TS were also serially diluted, spread on MRS, and incubated at 30, 20, and 10°C for 24‒48, 48‒72, and 144‒168 h, respectively. Subsequently, microbial counts were recorded (log CFU/ml). Next, approximately 30 colonies were chosen at random and subcultured in MRS agar. The isolates were identified by amplifying the 16S rRNA gene of bacteria using universal primers 27F (5¢-AGA GTT TGA TCM TGG CTC AG-3¢) and 1492R (5¢-TAC GGY TAC CTT GTT ACG ACT T-3¢).

### Next generation Sequencing (NGS)

DNA was extracted according to the manufacturer’s instructions using a DNeasyPowerSoil Kit (Qiagen, Germany). The extracted DNA was quantified using a Quant-IT PicoGreen assay kit (Invitrogen, USA). The sequencing libraries were prepared according to the Illumina 16S metagenomic sequencing library protocols to amplify the V3 and V4 regions. The first PCR product was purified using AMPure beads (Agencourt Bioscience, USA). Following purification, 2 μl of the first PCR product was amplified for final library construction containing the index using the NexteraXT indexed primer (Illumina, USA). The PCR product was purified using AMPure beads. Then, the final purified product was quantified using qPCR according to the qPCR quantification protocol guide (KAPA library quantification kits for Illumina sequencing platforms) and qualified using the TapeStation D1000 ScreenTape system (Agilent Technologies, Germany). Finally, the paired-end (2 × 300 bp) sequencing was performed through Macrogen systems (Korea) using the MiSeq platform (Illumina).

### Growth Curve of Kimchi LAB Isolated at Low Temperatures

To investigate the growth curve of LAB isolated at low isolation temperatures, including *Leu. gelidum* (from 4HSG, KCKM P0052), *Leu. gasicomitatum* (from 4TS, KCKM P0057), *Leu. inhae* (from 10HSG, KCKM P0061), *D. algida* (from 10YSD, KCKM P0083), *C. kimchiensis* (from 10YSD, KCKM P0044), and *W. koreensis* (from 4HSG, KCKM P0054), bacterial strains were incubated in MRS broth at 20°C for 24 h. Next, they were inoculated in MRS broth and incubated at 10, 15, 20, and 25°C. Each strain was incubated at 10 and 15°C for 96 h, or at 20 and 25°C for 72 h. The cultures were sampled every 24 h, serially diluted, spread on MRS agar, and incubated at 20°C for 48–72 h to count colony forming units. Finally, the corresponding growth curve was prepared.

### Bacterial Membrane Fatty Acid Composition

To determine the difference in thermal adaptation between *W. koreensis* that did not grow well at 30°C (KCKM P0035, KCKM P0054) and that which grew well at the same temperature (KCKM 0130), strains were cultured at different temperatures to analyze their membrane fatty acid composition. *W. koreensis* KCKM P0035 and *W. koreensis* KCKM P0054 were cultured at 10°C for 144‒168 h, 20°C for 48‒72 h, and 28°C for 24‒48 h. Similarly, *W. koreensis* KCKM 0130 was cultured at 20°C for 48‒72 h, 28°C for 24‒48 h, and 33°C for 24‒48 h. Next, cells were harvested using a 4 mm loop and stored at -80°C before analysis. These cells were analyzed according to the Sherlock Microbial Identification System (MIS) using the Agilent 6890N(G1530N) gas chromatograph and Sherlock version 6.1.

### Statistical Analysis

All physicochemical properties and bacterial counts of kimchi samples were analyzed in triplicates. In addition, SPSS v.27 (SPSS Inc, USA) was used to perform one-way analysis of variance and Duncan’s multiple range tests. Results are expressed as the mean ± standard deviation; *p* < 0.05 was considered statistically significant.

## Results

### Fermentation Conditions and Physicochemical Properties of Kimchi Samples

The fermentation conditions and physicochemical properties of kimchi samples are shown in Table 1. HM, KT 1 (initial fermentation phase), KT 2 (middle fermentation phase), and KT 3 (final fermentation phase) kimchi were used for LAB isolation. Moreover, 10INJ, 10TB, 10GSY, 10YSD, 10TS, 10HSG, 4INJ, 4TB, 4GSY, 4YSD, 4TS, and 4HSG were used to investigate appropriate temperature for the isolation of various LAB. The fermentation period and temperature range of all samples were 1‒250 days at 0‒10°C, respectively. The range of pH, titratable acidity, salinity, and soluble solid contents in kimchi samples were 4.01‒5.78, 0.42‒1.22%, 1.49‒2.40%, and 8.10‒10.20 °Brix, respectively.

Generally, pH in the initial fermentation phase was >5. However, it decreased to ≤4 after the late phase [11]. Further, the titratable acidity of the initial fermentation phase was below 0.4% and increased to >1% at the final phase. The changes in these physicochemical properties are indicative of the variety in fermentation conditions of the kimchi samples.

### Viable Cell Counts on Different Media and Isolation Temperatures

To investigate suitable culture media and temperatures, the LAB from HM, KT 1, KT 2, and KT 3 kimchi samples was evaluated under three types of LAB selective media and four isolation temperature conditions (Table 2). The temperature used to isolate LAB from kimchi was defined as the isolation temperature to avoid confusion with the culture or incubation temperature of the isolated strains. The LAB counts of HM, KT 1, KT 2, and KT 3 ranged from 7.44‒8.08 log CFU/ml, 6.17‒7.06 log CFU/ml, 8.52‒8.89 log CFU/ml, to 8.06‒8.58 log CFU/ml, respectively. The LAB counts in HM and KT 1 showed a slight difference depending on the isolation temperature. Furthermore, the highest LAB count was observed in MRS agar. Many components of culture medium can affect the growth of each strain, and MRS, LBS, and PES media have different compositions. These media contain compounds that promote LAB growth, such as carbohydrates, nitrogen sources, and Mg salt. MRS and LBS use dextrose as a carbon source. In contrast, PES is a selective medium for *Leuconostoc* sp. and uses sucrose because these species release dextransucrase in high-sucrose media. This enzyme subsequently converts sucrose into dextran [21]. The kinds of carbohydrates and nitrogen sources that promote growth in each strain differ. Thus, the growth of strains found in the kimchi samples could be promoted in a medium with a favored nutrient source [22]. These results demonstrate that MRS is the most suitable isolation medium.

### Viable Cell Counts at Different Isolation Temperatures

To investigate the suitable isolation temperature for various kimchi LAB, the number of colonies was counted at different isolation temperatures of 12 kimchi samples on MRS agar (Table 3). In only 4INJ and 4HSG, the number of colonies cultured at 20°C and 10°C was substantially higher than that at 30°C. As there was no significant difference between 10 of the 12 kimchi samples, analysis of the diversity of microorganisms isolated according to the isolation temperature was needed to determine the appropriate isolation temperature.

### Comparative Culture-Dependent and Culture-Independent Analyses

**Fig. 1:**
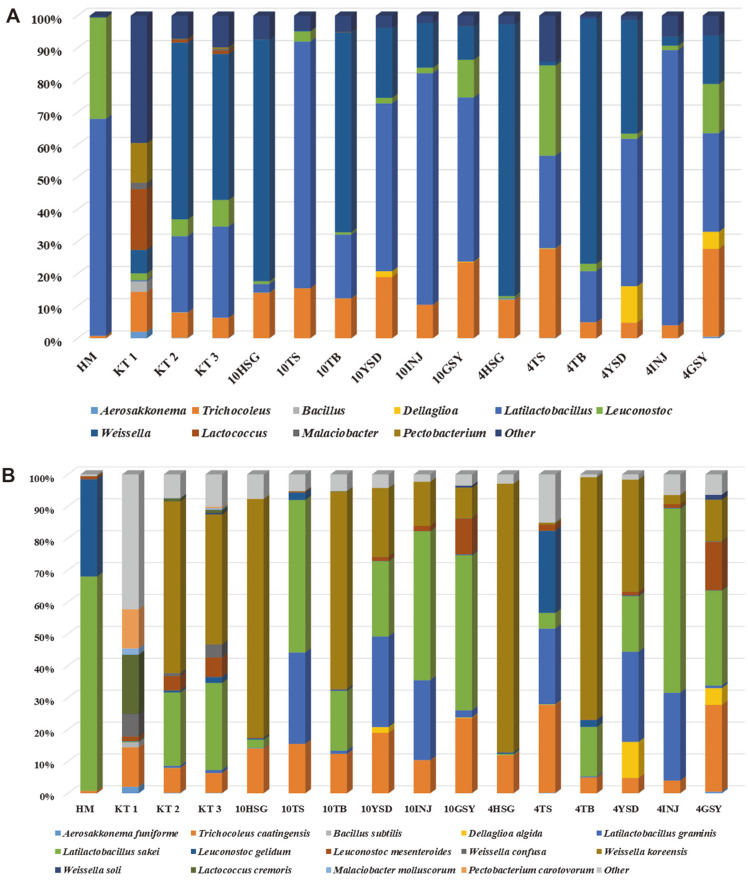
The relative abundances of microbes, as determined through metagenomic analysis. **A**: genus level; **B**: species level (representing >1%)

**Fig. 2:**
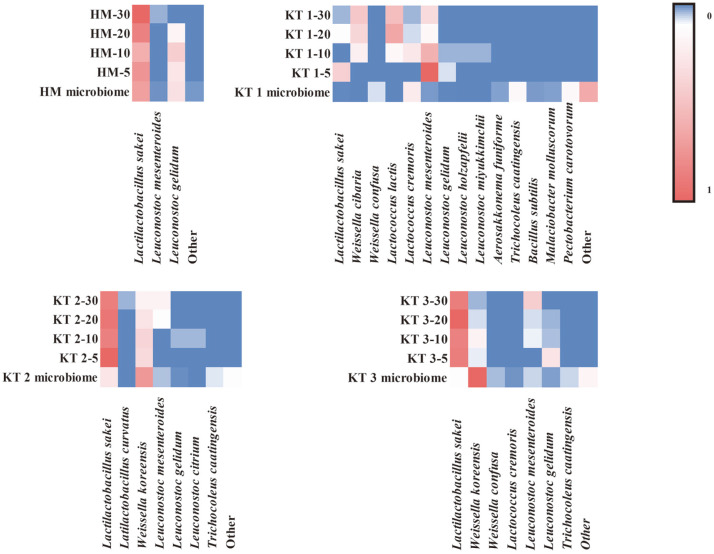
A heat map of the microbial community in culture-dependent and culture-independent analyses of four kimchi samples. The color intensity in each panel shows the percentage in the sample, according to the color key (right). The sample names are represented as isolation temperatures, and the result of culture-independent analysis is represented as the sample microbiome.

To determine the ideal isolation temperature for the LAB flora in kimchi, the culture-dependent and culture-independent results were compared. First, culture-independent analysis was performed. The results from kimchi samples are shown at genera and species levels in Fig. 1. As shown in Fig. 1A, *Latilactobacillus* sp. was the predominant genus in nine kimchi samples (HM, 10TS, 10YSD, 10INJ, 10GSY, 4TS, 4YSD, 4INJ, 4GSY) and *Weissella* sp. was the predominant genus in six samples (KT 2, KT 3, 10HSG, 10TB, 4HSG, 4TB). In KT 1, *Lactococcus* sp. was the predominant genus at the initial fermentation phase. However, other genera were also of similar relative abundance. Moreover, *Leuconostoc* sp. and *Trichocoleus* sp. showed the highest abundance in most of the samples. The species with the highest mean abundances were *W. koreensis* (30.52%), *L. sakei* (26.98%), *T. caatingensis* (12.62%), *L. graminis* (10.49%), *Leu. gelidum* (4.11%), *Leu. mesenteroides* (2.93%), *Lac. cremoris* (1.29%), and *D. algida* (1.18%) (Fig. 1B). During the culture-dependent analysis, 1,464 different LAB strains were isolated from kimchi samples. Information on strains isolated from kimchi at different temperatures is given in Tables S1–S16. Comparison of the diversity of 16 kimchi samples at different isolation temperatures was conducted using culture-dependent and culture-independent methods. To visualize the difference in the microbial community structure for some kimchi samples, relative abundance of species was represented in a heat-map style (Fig. 2). *L. graminis* was of high relative abundance in some kimchi samples (10YSD, 10INJ, 10GSY, 4YSD, and 4INJ) when analyzed through the culture-independent method, whereas it was not isolated by culture-dependent methods. This may be due to inappropriate culture conditions for this strain. The following LAB strains were not detected through the culture-independent method: *Lac. lactis*, *W. cibaria*, and *Leu. miyukkimchii* isolated using the culture-dependent method in KT 1; *Leu. Holzapfelii*; 10HSG, 10TS, and 10GSY, *L. curvatus*; 10HSG, 10TS, 10TB, 10YSD, 10INJ, 10GSY, 4YSD, 4INJ, and 4GSY *Leu. pseudomesenteroides*; *Leu. Inhae*; *Leu. falkenbergense*; *Companilactobacillus heilongjiangensis*; *Enterococcus casseliflavus*; *Leu. Lactis*; and *Leu. carnosum* isolated in KT 1; KT 2; 10HSG, 10TB, 10YSD, 10GSY, 4TS, and 4GSY; 10HSG, 4HSG, 4YSD, and 4GSY; 4HSG, 4TB, and 4GSY; 10HSG; 4HSG; 4TB; and 4GSY.

**Fig. 3:**
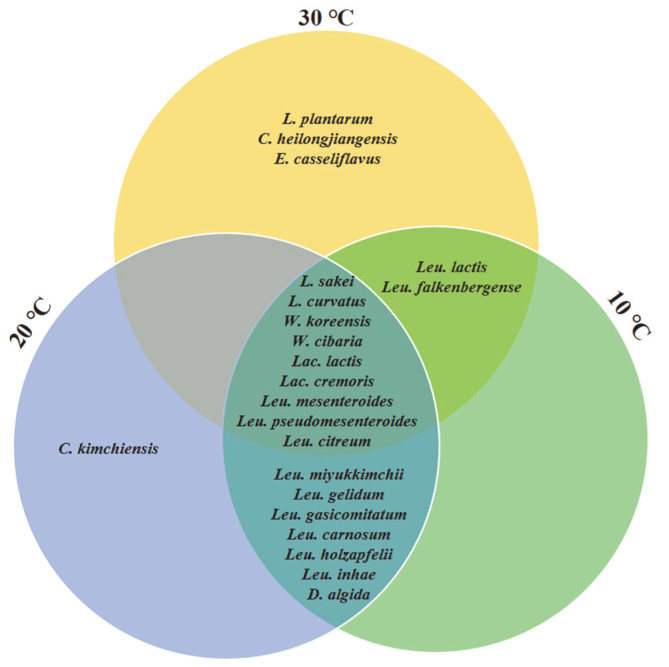
A Venn diagram of lactic acid bacteria isolated from 16 kimchi samples using a culture-dependent method based on isolation temperature.

Isolated strains and relative abundance were different depending on the isolation temperature. More strains from nine of the 16 kimchi samples were isolated at <30°C. Moreover, five samples showed minimal difference in the number of isolated strains at varying isolation temperatures. Contrastingly, more strains were isolated at 30°C in the remaining two samples. *Leu. gelidum*, *Leu. gasicomitatum*, *Leu. inhae*, *Dellaglioa algida*, *Companilactobacillus kimchiensis*, *Leu. miyukkimchii*, *Leu. holzapfelii* and *Leu. carnosum* were not isolated from all samples at 30°C (Fig. 3). Although *W. koreensis* generally grows well under mesophilic conditions [23], some strains did not grow well at 30°C.

### Cultural Characteristics of Psychrotrophic Strains

**Fig. 4:**
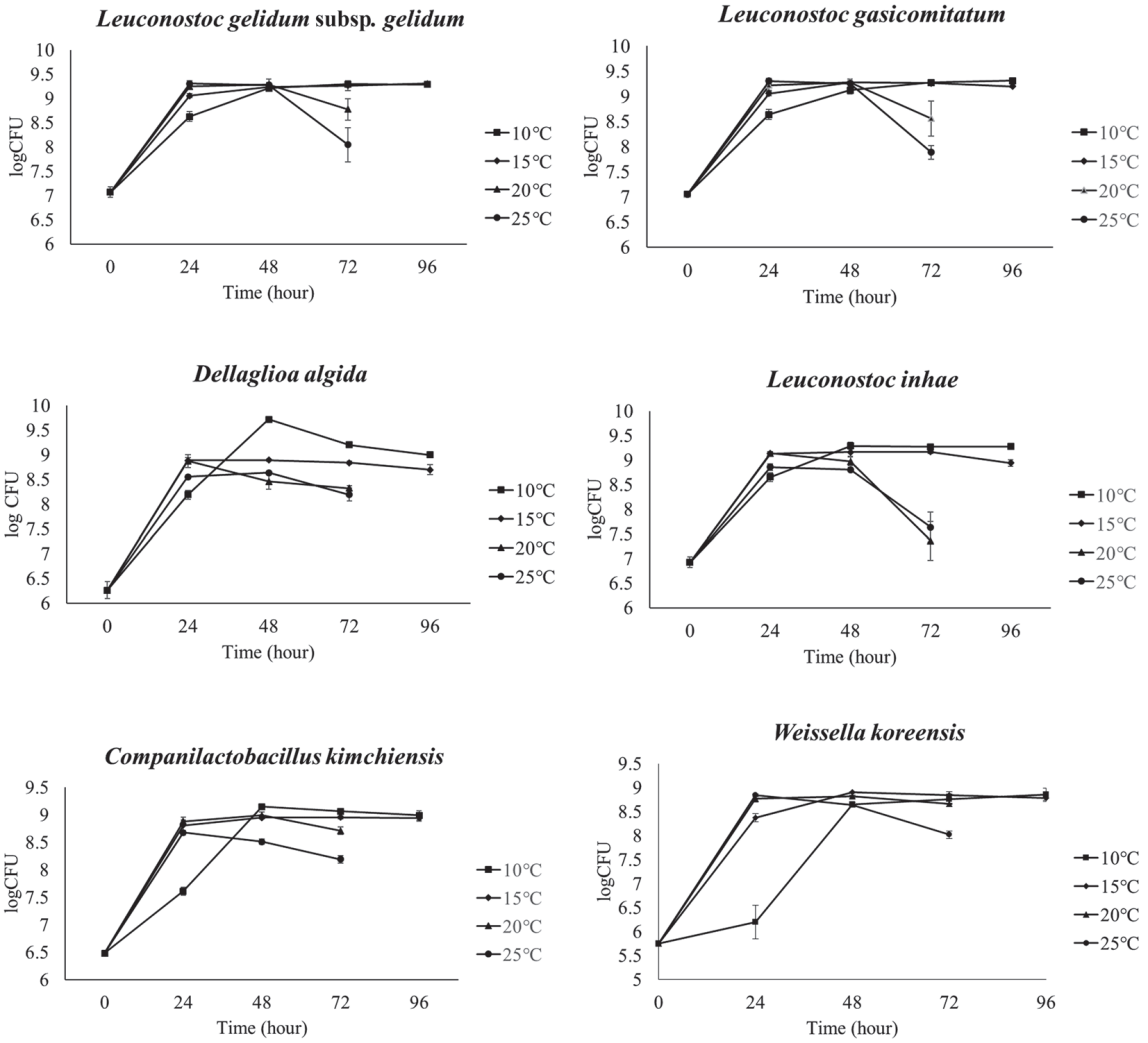
The growth curve of six selected strains at different culture temperatures (10, 15, 20, and 25°C).

The culture characteristics of psychrotrophic kimchi LAB strains that were isolated at low temperatures and did not grow well at 30°C were investigated. *Leu. gelidum* (KCKM P0052), *Leu. gasicomitatum* (KCKM P0057), *Leu. Inhae* (KCKM P0061), *D. algida* (KCKM P0083), *C. kimchiensis* (KCKM P0044), and *W. koreensis* (KCKM P0054) were cultured at 10, 15, 20, and 25°C (Fig. 4). The growth curves of these isolates showed good growth at all the aforementioned temperatures, confirming their psychrotrophic characteristics.

### Membrane Fatty Acid Composition of *W. koreensis*

Among the strains isolated at 10, 20, and 30°C, *W. koreensis* was divided into strains that grew well at 30°C (KCKM 0130) and those that did not grow (KCKM P0035) or slightly grew (KCKM P0054). Thus, to determine the difference in cultural characteristics between psychrotrophic and mesophilic *W. koreensis*, we compared changes in membrane fatty acid composition according to culture temperature of isolates with low isolation temperature (KCKM P0035 and KCKM P0054) and mesophilic strains (KCKM 0130) (Table 4). There was no specific trend in the membrane fatty acid composition of *W. koreensis* KCKM P0035 as the culture temperature increased. Contrastingly, the membrane fatty acid composition of *W. koreensis* KCKM 0130 changed with increasing culture temperature. Similarly, the C16:0, C16:1 ω7c/C16:1 ω6c ratio increased as the culture temperature increased. However, C14:0, C18:1 ω9c, and C19:0 decreased with increasing culture temperature. Moreover, as the culture temperature increased, the saturated fatty acid ratio increased, whereas the unsaturated fatty acid ratio decreased. *W. koreensis* KCKM P0054 showed a similar trend to *W. koreensis* KCKM 0130. However, the ratio of unsaturated fatty acids was almost unchanged as the temperature increased.

## Discussion

This study investigated the appropriate conditions for isolating diverse LAB species from kimchi. Further, the cultural characteristics of psychrotrophs that did not grow well at medium temperature (30°C) were investigated. The diversity of LAB differed based on the isolation temperature of kimchi samples. In particular, *Leu. gelidum*, *Leu. gasicomitatum*, *Leu. inhae*, *D. algida*, *C. kimchiensis*, *Leu. miyukkimchii*, *Leu. holzapfelii*, and *Leu. carnosum* could not be isolated at 30°C. These findings indicate that 30°C is an unsuitable temperature for *C. kimchiensis*, *Leu. miyukkimchii*, and *Leu. carnosum* growth, despite the known optimum cultural temperature being approximately 30°C [23-25]. In a previous study, the appropriate cultural temperature of *Leu. cremoris* was 24‒27°C [26]. Additionally, *Leu. gelidum* and *D. algida* are typical psychrotrophic LAB [27, 28]. *Leu. inhae* also could not grow at 30°C in previous studies [7, 29]. *Leuconostoc* spp. can survive under cold stress conditions by expressing heat stress proteins [30]. *Weissella* spp. can also grow at low temperatures, and *W. serratia* growth is inhibited at >15°C [31]. Further, Mäkelä *et al*. [32] attempted to isolate ropy slime-forming strains, namely *Lactobacillus* spp. and *Leuconostoc* spp., at 30°C, 20°C, and 15°C. Most ropy slime forming strains were detected at 20°C and 15°C. Thus, the isolation temperature at 30°C seemed unsuitable for isolation of these ropy colony strains. Therefore, it may be inappropriate to isolate phychrotrophic LAB from kimchi under only mesophilic conditions (30°C).

Some LAB strains, such as *Lac. lactis* and *L. plantarum* were not isolated at 5°C. Furthermore, the culture period (14 days) was delayed at 5°C compared to that of conditions maintained at other isolation temperatures. These results showed low efficiency of 5°C in strain incubation. The suitable growth range for *Lac. lactis* is 27‒33°C [33, 34]. Therefore, 5°C was not a suitable isolation temperature for the diverse LAB from kimchi. The results of culture-dependent analysis obtained at 10°C and 20°C were considered more similar to culture-independent assay results than those obtained at 5°C and 30°C. This study found that 10°C and 20°C are suitable temperatures for isolating various psychrotrophic LAB, owing to growth retardation of strains at 5°C and inhibition of psychrotrophic LAB growth at 30°C.

Some LAB, including *W. confusa*, were identified using the culture-independent method but could not be identified through culture-dependent analysis. This is because the culture-independent method can detect the nucleotide sequences of dead microbiota with intact cells or those not cultured in the medium [35]. In contrast, some strains, including *W. cibaria* and *Lac. Lactis*, were identified through culture-dependent methods rather than the culture-independent method. Similar to our results, previous studies reported that some populations were missed by culture-independent methods due to their low numbers, which might be increased by culture-dependent approaches [36-38]. In the culture-dependent results, some of the *Leuconostoc* spp. were isolated at 10°C and 20°C whereas *L. plantarum*, *C. heilongjiangensis*, and *E. casseliflavus* were isolated only at 30°C. *L. plantarum* is a mesophilic bacterium [39, 40]. Therefore, it may have been isolated only at 30°C. As these strains were isolated only once from all samples, more samples are needed to confirm the isolation temperature of these strains. In this study, some mesophilic strains, including *W. koreensis*, *Leu. carnosum*, *C. kimchiensis*, and *Leu. miyukkimchii*, did not grow well at 30°C. Therefore, the difference in thermal tolerance between these strains must be investigated. The membrane fatty acid composition and adaptation of LAB under stress conditions are closely related [41]. For example, increased thermal tolerance was seen with the decrease of unsaturated fatty acids or the increase of saturated fatty acids in bacterial cell membrane fatty acids [41, 42]. In this study, an increased culture temperature of *W. koreensis* resulted in a decrease in the unsaturated fatty acid ratio of the thermal resistant strain. However, there was no difference in the strain with low thermal resistance. Furthermore, the saturated fatty acid ratio was increased with culture temperature in thermal resistant strains. An increased saturated fatty acid ratio or a decreased unsaturated fatty acid ratio enhances the thermal resistance of bacteria [41, 43]. In our study, *W. koreensis* KCKM 0130, whose growth limit was >30°C, could have decreased the unsaturated fatty acid content while increasing the saturated fatty acid content to adapt to the high culture temperature. Moreover, low thermal adaptation of *W. koreensis* KCKM P0035 and *W. koreensis* KCKM P0054, whose growth limit was <30°C, was possibly due to the low metabolism related to the change in membrane fatty acid composition. A medium temperature (approximately 30°C) is typically applied to isolate LAB from kimchi. However, more diverse strains were isolated at the isolation temperatures of 10°C and 20°C than at 30°C from most kimchi samples in this study. This indicates that only 30°C is insufficient for the isolation of more diverse LAB from kimchi.

Unlike kimchi, some fermented vegetables are fermented at room temperature using fewer ingredients [44, 45]. Therefore, kimchi fermented at temperatures below 10°C using various ingredients is expected to contain diverse psychrotrophic lactic acid bacteria.

Additionally, most psychrotrophic LAB isolated in this study may be key players in kimchi fermentation, with *Leu. gelidum* as one of the dominant strains [1]. However, recent studies suggest that psychrotrophic LAB, particularly *Leu. gelidum*, are food spoilage organisms that reduce the quality of fermented foods [18, 27, 46]. Thus, it is necessary to further investigate the influence of psychrotrophic strains on kimchi fermentation.

Our results suggest that the culture media composition, incubation time, and temperature in the isolation procedures are important for isolating diverse LAB from kimchi. Hence, mesophilic conditions (approximately 30°C) and isolation temperatures of 10–20°C in MRS medium should be used in parallel to isolate diverse LAB, including psychrotrophs, from kimchi. These findings can contribute to the isolation of more diverse LAB strains from kimchi.

## Supplemental Materials

Supplementary data for this paper are available on-line only at http://jmb.or.kr.

## Figures and Tables

**Table 1:** The fermentation conditions and physicochemical properties of kimchi samples.

Sample	Fermentation period (day)	Fermentation temperature (°C)	pH	Titratable acidity (%)	Salinity (%)	Soluble solid contents (°Brix)
HM	250	0 ± 2	4.85 ± 0.02	0.72 ± 0.04	1.89 ± 0.01	10.17 ± 0.15
KT 1	1	4 ± 1	5.78 ± 0.06	0.42 ± 0.00	1.49 ± 0.14	10.20 ± 0.61
KT 2	15	4 ± 1	4.32 ± 0.04	1.06 ± 0.04	1.70 ± 0.05	10.00 ± 0.30
KT 3	29	4 ± 1	4.07 ± 0.10	1.22 ± 0.03	1.60 ± 0.09	8.43 ± 0.12
10HSG	7	10 ± 1	4.40 ± 0.01	0.92 ± 0.05	1.87 ± 0.08	8.20 ± 0.36
10TS	7	10 ± 1	4.13 ± 0.03	0.90 ± 0.04	1.62 ± 0.09	9.30 ± 0.30
10TB	7	10 ± 1	4.16 ± 0.02	0.91 ± 0.04	2.15 ± 0.09	8.17 ± 0.31
10YSD	7	10 ± 1	4.01 ± 0.01	0.93 ± 0.01	2.40 ± 0.05	8.43 ± 0.32
10INJ	7	10 ± 1	4.14 ± 0.04	0.91 ± 0.04	1.77 ± 0.02	9.34 ± 0.62
10GSY	7	10 ± 1	4.25 ± 0.01	0.96 ± 0.02	1.65 ± 0.01	8.17 ± 0.47
4HSG	14	4 ± 1	4.40 ± 0.02	0.88 ± 0.07	1.61 ± 0.06	8.37 ± 0.40
4TS	14	4 ± 1	4.22 ± 0.02	0.88 ± 0.03	1.54 ± 0.07	9.73 ± 0.49
4TB	14	4 ± 1	4.31 ± 0.01	0.77 ± 0.03	1.74 ± 0.07	7.53 ± 0.25
4YSD	14	4 ± 1	4.13 ± 0.03	0.78 ± 0.04	2.06 ± 0.02	8.10 ± 0.46
4INJ	14	4 ± 1	4.27 ± 0.03	0.83 ± 0.02	1.64 ± 0.03	9.37 ± 0.31
4GSY	14	4 ± 1	4.48 ± 0.02	0.93 ± 0.06	1.63 ± 0.06	9.87 ± 0.46

**Table 2:** Bacterial count of four kimchi samples using different culture media.

Sample	Temperature (°C)	Count (log CFU/ml)			
		PCA	MRS	PES	LBS
HM	30	7.42 ± 0.05	7.60 ± 0.03^a^	7.44 ± 0.06^a^	7.51 ± 0.05^a^
	20		7.91 ± 0.03^b^	7.93 ± 0.04^b^	7.49 ± 0.06^a^
	10		8.04 ± 0.09^c^	7.87 ± 0.03^b^	7.64 ± 0.03^b^
	5		8.08 ± 0.10^c^	7.93 ± 0.06^b^	7.54 ± 0.03^a^
KT1	30	7.06 ± 0.09	7.02 ± 0.15^b^	6.99 ± 0.08^b^	6.40 ± 0.05^b^
	20		7.06 ± 0.12^b^	7.03 ± 0.09^b^	6.43 ± 0.12^b^
	10		7.06 ± 0.03^b^	6.96 ± 0.10^b^	6.22 ± 0.09^a^
	5		6.19 ± 0.16^a^	6.24 ± 0.15^a^	6.17 ± 0.10^a^
KT2	30	8.78 ± 0.06	8.85 ± 0.05^a^	8.82 ± 0.08^a^	8.64 ± 0.08^b^
	20		8.87 ± 0.02^a^	8.75 ± 0.02^a^	8.52 ± 0.04^a^
	10		8.87 ± 0.01^a^	8.82 ± 0.09^a^	8.59 ± 0.02^ab^
	5		8.89 ± 0.01^a^	8.88 ± 0.05^a^	8.53 ± 0.04^a^
KT3	30	8.22 ± 0.14	8.56 ± 0.05^a^	8.44 ± 0.06^a^	8.29 ± 0.11^b^
	20		8.52 ± 0.09^a^	8.43 ± 0.13^a^	8.51 ± 0.04^c^
	10		8.58 ± 0.05^a^	8.52 ± 0.05^a^	8.20 ± 0.15^ab^
	5		8.48 ± 0.02^a^	8.43 ± 0.07^a^	8.06 ± 0.06^a^

**Table 3:** Lactic acid bacteria count of 12 kimchi samples at different isolation temperatures.

Sample	Temperature (°C)		
	30	20	10
10INJ	8.86 ± 0.22^a^	9.03 ± 0.03^a^	8.96 ± 0.11^a^
10TB	8.95 ± 0.04^a^	8.91 ± 0.08^a^	8.95 ± 0.02^a^
10GSY	9.03 ± 0.09^a^	9.14 ± 0.06^a^	9.12 ± 0.07^a^
10YSD	8.68 ± 0.03^a^	8.78 ± 0.07^a^	8.79 ± 0.09^a^
10TS	9.23 ± 0.07^a^	9.25 ± 0.03^a^	9.19 ± 0.05^a^
10HSG	8.68 ± 0.17^a^	8.61 ± 0.30^a^	8.92 ± 0.11^a^
4INJ	8.75 ± 0.05^a^	8.87 ± 0.01^b^	8.86 ± 0.05^b^
4TB	8.35 ± 0.31^a^	8.50 ± 0.15^a^	8.56 ± 0.10^a^
4GSY	8.91 ± 0.03^a^	8.99 ± 0.02^a^	8.93 ± 0.16^a^
4YSD	8.22 ± 0.16^a^	8.62 ± 0.47^a^	8.39 ± 0.02^a^
4TS	8.90 ± 0.08^a^	9.08 ± 0.07^a^	9.03 ± 0.15^a^
4HSG	8.19 ± 0.02^a^	8.58 ± 0.08^b^	9.42 ± 0.13^c^

**Table 4:** Comparison of relative membrane fatty acid composition between mesophilic and psychrotrophic *Weissella koreensis* at different culture temperatures.

	KCKM P0035			KCKM P0054			KCKM 0130		
Fatty acid (%)	10°C	20°C	28°C	10°C	20°C	28°C	20°C	28°C	33°C
Saturated									
C_12:0_	1.06±0.06	0.69±0.06	1.20±0.11	2.00±0.03	2.05±0.27	1.49±0.43	1.97±0.32	2.56±0.39	1.92±0.39
C_14:0_	1.18±0.02	1.21±0.08	1.22±0.02	1.75±0.02	1.33±0.05	1.32±0.02	1.45±0.08	1.45±0.14	1.21±0.16
C_16:0_	10.68±0.11	11.29±0.05	10.51±0.40	9.63±0.11	10.54±0.12	13.22±0.12	10.80±0.22	10.81±0.41	16.53±0.82
C_17:0_		0.19±0.03							
C_18:0_	1.40±0.05	1.18±0.02	1.49±0.09	1.54±0.02	1.61±0.06	1.06±0.09	1.44±0.04	1.59±0.07	0.96±0.24
Unsaturated									
C_18:1_ ω9c	80.40±0.25	80.10±0.54	80.36±0.28	78.90±0.54	78.85±1.07	78.70±0.85	78.99±0.69	76.92±1.21	75.19±1.74
C_18:3_ ω6c							0.39±0.04	0.46±0.06	0.38±0.11
Branched-chain fatty acid									
C_19:0_ iso	2.78±0.05	2.48±0.28	2.74±0.29	3.10±0.09	2.95±0.27	1.72±0.05	3.44±0.20	3.20±0.15	1.63±0.58
Hydroxy fatty acids									
C_17:0_ 2OH	2.07±0.09	1.75±0.03	1.96±0.16	2.64±0.07	2.00±0.16	1.29±0.13	1.12±0.19	1.32±0.11	1.05±0.41
C_16:1_ 2OH								0.63±1.10	
Summed feature									
C_16:1_ ω7c/C_16:1_ ω6c	0.42±0.16	1.10±0.88	0.77±0.13	0.65±0.17	0.99±0.34	1.20±0.56	0.40±0.70	1.08±0.96	1.88±0.42
Total saturated fatty acid	14.32±0.11	14.56±0.18	14.42±0.22	14.93±0.08	15.53±0.48	17.08±0.41	15.66±0.60	16.41±0.95	20.62±1.06
^a^Total unsaturated fatty acid	80.82±0.09	81.21±0.37	80.87±0.19	79.34±0.15	79.51±0.61	79.91±0.31	79.78±0.61	78.46±1.02	76.69±0.86

^a^Total unsaturated fatty acid: unsaturated + C_16:1_ ω7c/C_16:1_ ω6c
