# Supplementary material for: Influence of Isolation Temperature on Isolating Diverse Lactic Acid Bacteria from Kimchi and Cultural Characteristics of Psychrotrophs
Source: J Microbiol Biotechnol. 2023 May 12;33(8):1066–75. doi: 10.4014/jmb.2303.03047 (PMC10468671; doi:10.4014/jmb.2303.03047)
Supplement: Supplementary file 1 [file jmb-33-8-1066-supple.pdf]

J. Microbiol. Biotechnol.
https://doi.org/10.4014/jmb.2303.03047

Supplementary Tables

Supplementary Table 1. Number of bacteria isolated from HM at different temperatures.

Strain	Culture temperature (°C)			
	30	20	10	5
Lactilactobacillus sakei	30	24	16	16
Leuconostoc mesenteroides	1	0	0	0
Leuconostoc gelidum	0	5	11	6
Total	31	29	27	22

Supplementary Table 2. Number of bacteria isolated from KT 1 at different temperatures.

Strain	Culture temperature (°C)			
	30	20	10	5
Lactilactobacillus sakei	1	3	0	8
Weissella cibaria	9	8	5	0
Lactococcus lactis	10	13	4	0
Lactococcus cremoris	1	2	6	0
Leuconostoc mesenteroides	7	4	12	18
Leuconostoc gelidum	0	0	1	2
Leuconostoc holzapfelii	0	0	1	0
Leuconostoc miyukkimchii	0	0	1	0
Total	28	30	30	28

Supplementary Table 3. Number of bacteria isolated from KT 2 at different temperatures.

Strain	Culture temperature (°C)			
	30	20	10	5
Lactilactobacillus sakei	19	20	17	21
Latilactobacillus curvatus	1	0	0	0
Weissella koreensis	5	7	8	8
Leuconostoc mesenteroides	5	3	0	0
Leuconostoc gelidum	0	0	1	0
Leuconostoc citreum	0	0	1	0
Total	30	30	27	29

Supplementary Table 4. Number of bacteria isolated from KT 3 at different temperatures.

Strain	Culture temperature (°C)			
	30	20	10	5
Lactilactobacillus sakei	21	24	17	18
Weissella koreensis	1	2	3	2
Leuconostoc mesenteroides	6	2	2	0
Leuconostoc gelidum	0	1	1	4
Total	28	29	23	24

Supplementary Table 5. Number of bacteria isolated from 10INJ at different temperatures.

Strain	Culture temperature (°C)		
	30	20	10
Lactilactobacillus sakei	16	13	10
Latilactobacillus curvatus	8	5	12
Weissella koreensis	1	10	7
Leuconostoc mesenteroides	4	2	1
Leuconostoc citreum	1	0	0
Total	30	30	30

Supplementary Table 6. Number of bacteria isolated from 10TB at different temperatures.

Strain	Culture temperature (°C)		
	30	20	10
Lactilactobacillus sakei	13	9	12
Latilactobacillus curvatus	4	1	2
Weissella koreensis	10	18	10
Lactococcus lactis	1	0	0
Leuconostoc mesenteroides	1	0	0
Leuconostoc pseudomesenteroides	0	0	1
Leuconostoc lactis	1	0	1
Leuconostoc gelidum	0	0	4
Leuconostoc citreum	0	2	0
Total	30	30	30

Supplementary Table 7. Number of bacteria isolated from 10GSY at different temperatures.

Strain	Culture temperature (°C)		
	30	20	10
Lactilactobacillus sakei	8	3	6
Latilactobacillus curvatus	2	2	3
Weissella koreensis	4	5	6
Leuconostoc mesenteroides	12	12	11
Leuconostoc pseudomesenteroides	0	1	3
Leuconostoc gelidum	0	2	0
Leuconostoc citreum	3	1	0
Leuconostoc holzapfelii	0	0	1
Total	29	26	30

Supplementary Table 8. Number of bacteria isolated from 10YSD at different temperatures.

Strain	Culture temperature (°C)		
	30	20	10
Lactilactobacillus sakei	9	11	7
Latilactobacillus curvatus	9	8	8
Weissella koreensis	5	4	10
Leuconostoc mesenteroides	3	2	1
Leuconostoc pseudomesenteroides	0	1	0
Leuconostoc gelidum	0	1	0
Leuconostoc carnosum	0	1	2
Companilactobacillus kimchiensis	0	1	0
Dellaglioa algida	0	1	2
Total	26	30	30

Supplementary Table 9. Number of bacteria isolated from 10TS at different temperatures.

Strain	Culture temperature (°C)		
	30	20	10
Lactilactobacillus sakei	17	11	12
Latilactobacillus curvatus	10	9	10
Leuconostoc mesenteroides	1	1	4
Leuconostoc pseudomesenteroides	0	1	0
Leuconostoc gelidum	0	6	0
Leuconostoc holzapfelii	0	2	0
Leuconostoc citreum	2	0	0
Total	30	30	26

Supplementary Table 10. Number of bacteria isolated from 10HSG at different temperatures.

Strain	Culture temperature (°C)		
	30	20	10
Lactilactobacillus sakei	1	2	1
Latilactobacillus curvatus	0	1	0
Lactiplantibacillus plantarum	1	0	0
Weissella koreensis	24	22	16
Leuconostoc mesenteroides	2	0	0
Leuconostoc pseudomesenteroides	1	0	0
Leuconostoc gelidum	0	1	6
Leuconostoc holzapfelii	0	1	0
Leuconostoc citreum	1	0	0
Leuconostoc inhae	0	1	1
Companilactobacillus heilongjiangensis	1	0	0
Total	31	28	24

Supplementary Table 11. Number of bacteria isolated from 4INJ at different temperatures.

Strain	Culture temperature (°C)		
	30	20	10
Lactilactobacillus sakei	20	15	13
Latilactobacillus curvatus	3	5	6
Weissella koreensis	4	7	9
Leuconostoc mesenteroides	2	2	0
Total	29	29	28

Supplementary Table 12. Number of bacteria isolated from 4TB at different temperatures.

Strain	Culture temperature (°C)		
	30	20	10
Lactilactobacillus sakei	14	19	23
Weissella koreensis	0	0	2
Leuconostoc falkenbergense	1	0	0
Leuconostoc mesenteroides	5	2	0
Leuconostoc lactis	0	0	2
Leuconostoc gelidum	0	6	1
Leuconostoc citreum	1	0	0
Total	21	27	28

Supplementary Table 13. Number of bacteria isolated from 4GSY at different temperatures.

Strain	Culture temperature (°C)		
	30	20	10
Lactilactobacillus sakei	3	4	2
Latilactobacillus curvatus	1	1	0
Weissella koreensis	13	10	21
Leuconostoc mesenteroides	11	11	4
Leuconostoc pseudomesenteroides	1	0	0
Leuconostoc gelidum	0	2	2
Leuconostoc carnosum	0	1	0
Leuconostoc falkenbergense	0	0	1
Leuconostoc inhae	0	0	1
Total	29	29	31

Supplementary Table 14. Number of bacteria isolated from 4YSD at different temperatures.

Strain	Culture temperature (°C)		
	30	20	10
Lactilactobacillus sakei	16	10	11
Latilactobacillus curvatus	9	3	0
Weissella koreensis	3	6	0
Leuconostoc mesenteroides	1	1	1
Leuconostoc inhae	0	3	0
Leuconostoc carnosum	0	1	0
Dellaglioa algida	0	0	11
Total	29	24	23

Supplementary Table 15. Number of bacteria isolated from 4TS at different temperatures.

Strain	Culture temperature (°C)		
	30	20	10
Lactilactobacillus sakei	20	8	14
Latilactobacillus curvatus	10	2	4
Weissella koreensis	0	1	0
Leuconostoc mesenteroides	0	3	1
Leuconostoc pseudomesenteroides	0	1	0
Leuconostoc gelidum	0	12	10
Leuconostoc miyukkimchii	0	3	1
Total	30	30	30

Supplementary Table 16. Number of bacteria isolated from 4HSG at different temperatures.

Strain	Culture temperature (°C)		
	30	20	10
Lactilactobacillus sakei	0	0	1
Weissella koreensis	19	14	13
Leuconostoc mesenteroides	5	2	0
Leuconostoc gelidum	0	8	9
Leuconostoc falkenbergense	1	0	0
Leuconostoc carnosum	0	3	0
Leuconostoc citreum	0	1	1
Leuconostoc inhae	0	2	2
Enterococcus casseliflavus	1	0	0
Total	26	30	26
